# Oocytes maintain low ROS levels to support the dormancy of primordial follicles

**DOI:** 10.1111/acel.14338

**Published:** 2024-09-19

**Authors:** Shaogang Qin, Xinyue Chi, Zijian Zhu, Chuanhe Chen, Tuo Zhang, Meina He, Meng Gao, Ting Zhao, Jingwen Zhang, Lifan Zhang, Wenying Zheng, Ziqi Chen, Wenji Wang, Bo Zhou, Guoliang Xia, Chao Wang

**Affiliations:** ^1^ State Key Laboratory of Farm Animal Biotech Breeding College of Biological Sciences, China Agricultural University Beijing China; ^2^ State Key Laboratory of Animal Nutrition Institute of Animal Science, Chinese Academy of Agricultural Sciences Beijing China; ^3^ Guizhou Provincial Key Laboratory of Pathogenesis and Drug Research on Common Chronic Diseases, Department of Physiology College of Basic Medicine, Guizhou Medical University Guiyang Guizhou Province China; ^4^ Key Laboratory of Ministry of Education for Conservation and Utilization of Special Biological Resources in the Western China College of Life Science, Ningxia University Yinchuan China; ^5^ School of Life Science, Taizhou University Taizhou China

**Keywords:** ferroptosis, primary ovarian insufficiency, primordial follicle, ROS, SOD1

## Abstract

Primordial follicles (PFs) function as the long‐term reserve for female reproduction, remaining dormant in the ovaries and becoming progressively depleted with age. Oxidative stress plays an important role in promoting female reproductive senescence during aging, but the underlying mechanisms remain unclear. Here, we find that low levels of reactive oxygen species (ROS) are essential for sustaining PF dormancy. Compared to growing follicles, oocytes within PFs were shown to be more susceptible to ROS, which accumulates and damages PFs to promote reproductive senescence. Mechanistically, oocytes within PFs were shown to express high levels of the intracellular antioxidant enzyme superoxide dismutase 1 (SOD1), counteracting ROS accumulation. Decreased SOD1 expression, as a result of aging or through the experimental deletion of the *Sod1* gene in oocytes, resulted in increased oxidative stress and triggered ferroptosis within PFs. In conclusion, this study identified antioxidant defense mechanisms protecting PFs in mouse ovaries and characterized cell death mechanisms of oxidative stress‐induced PF death.

## Graphical abstract

Oocyte in primordial follicle of young mice exhibit higher expression of antioxidant enzymes, maintaining oocyte ROS at lower levels. With increasing age, the expression of SOD1 in NGO declines, leading to ROS accumulation, triggering primordial follicle loss due to ferroptosis, subsequently resulting in ovarian aging.

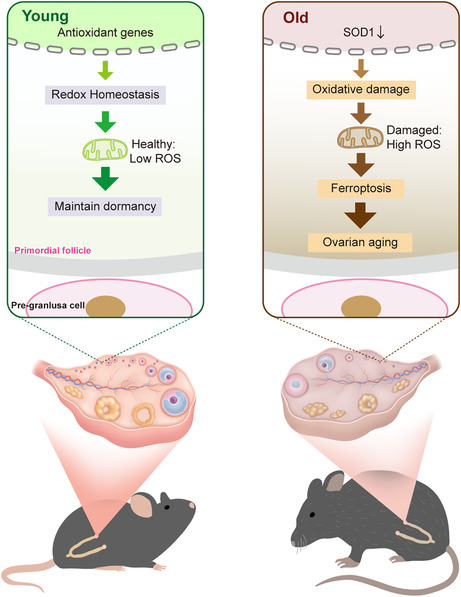

**Abbreviations**
ACSL4: Fatty Acid‐CoA Ligase 4
COXIV: Cytochrome C Oxidase Subunit IV
CUT & Tag: Cleavage under Targets and Tagmentation
DEGs: Differentially Expressed Genes
DHE: Dihydroethidium
ECM: Extracellular Matrix
FOXL2: Forkhead Box L2
FOXO3: Forkhead Box O3
GOs: Growing Oocytes
GPx: Glutathione Peroxidase
GSEA: Gene Set Enrichment Analysis
HO‐1: Heme Oxygenase‐1
KEGG: Kyoto Encyclopedia of Genes and Genomes
NAC: N‐Acetyl Cysteine
NGOs: Non‐growing Oocytes
NQO1: NAD(P)H Quinone Oxidoreductase 1
OS: Oxidative Stress
OXPHOS: Oxidative Phosphorylation
PFs: Primordial Follicles
PI3K‐AKT: Phosphoinositide 3‐kinase‐Protein Kinase B
POI: Primary Ovarian Insufficiency
PRDX3: Peroxiredoxin 3
PRDX3: Peroxiredoxin 3
ROS: Reactive Oxygen Species
SOD1: Cu‐Zn Superoxide Dismutase

## 1 INTRODUCTION

Primordial follicles (PFs) are the foundation of follicular development in female mammals, serving as the only source of growing follicles and ova (Adhikari & Liu, 2009). Established in perinatal ovaries, most PFs remain dormant until they are either activated or degenerate (Hamazaki et al., 2021; Zhang & Liu, 2015). Adult ovaries lack germline stem cells, making follicles a nonrenewable resource that is gradually depleted throughout life, resulting in reproductive senescence. Premature PF depletion can result in primary ovarian insufficiency (POI), affecting reproductive health and causing infertility in women of childbearing age (Zhang et al., 2014). POI is marked by irregular menstruation, decreased estrogen, increased gonadotropin levels, and is associated with health risks such as cardiovascular diseases and osteoporosis (Stuenkel & Gompel, 2023). Thus, maintaining PF dormancy is critical. Despite existing studies on the signaling mechanisms sustaining PFs, there is still a significant gap our knowledge of factors influencing PF development and promote their decline during aging. Further exploration is essential to uncover these influences and unravel the mysteries of PF aging.

Levels of reactive oxygen species (ROS) are indicators of ovarian aging that increase as the ovaries mature (Bao et al., 2023; Smits et al., 2023). While ROS play roles in essential reproductive processes such as oocyte maturation, fertilization, and embryonic development (Wang et al., 2021), excessive ROS results in oxidative stress (OS) which damages cells, promotes inflammation through the NF‐κB and inflammasome pathways, and disrupts metabolism and epigenetic regulation (Forrester et al., 2018; Snieckute et al., 2023; Wu et al., 2023). In the ovaries, elevated ROS levels can damage oocyte chromosomes, inducing spindle defects and meiotic instability that inhibit successful fertilization and promote reproductive disorders including polycystic ovary syndrome and POI (Das & Destouni, 2023; Guo et al., 2023). ROS also triggers pathways leading to follicular atresia, with high ROS levels in neonatal ovaries potentially causing apoptosis and affecting PF pool establishment (Wang, Liu et al., 2022 Wang, Zhang et al., 2022). The in vivo antioxidant defense system, comprising enzymes like superoxide dismutase (SOD), catalase (CAT), glutathione peroxidase (GPx), thioredoxin reductase (TrxR), heme oxygenase‐1 (HO‐1), and NAD(P)H quinone oxidoreductase 1 (NQO1), along with nonenzymatic components, regulates levels of ROS (Liang et al., 2023). However, the antioxidant capacity of these systems diminishes with age. Supplementation with substances like glutathione, selenium, vitamin C, resveratrol, coenzyme Q10, and melatonin has been shown to mitigate OS (Liang et al., 2023; Lim & Luderer, 2011; Secomandi et al., 2022). SOD1, a key intracellular oxidant, converts harmful superoxide radicals into hydrogen peroxide, which is further neutralized into water and oxygen (Fukai & Ushio‐Fukai, 2011). Female mice lacking SOD1 exhibit signs of accelerated reproductive aging including lower pregnancy rates, smaller litter sizes, and reduced corpus luteum size and progesterone levels (Matzuk et al., 1998; Noda et al., 2012).

Aging in females is characterized by an imbalance of ROS production and ROS neutralization, leading to ROS accumulation and deteriorated ovarian function. Research indicates that expression of antioxidant genes, including GPX1 and GSR, is much lower in early‐stage follicle oocytes compared to those in later stages, suggesting increased susceptibility of early‐stage oocytes to OS associated with aging (Wang et al., 2020). Deletion of *Gclm*, a critical mediator of glutathione synthesis in mice, results in decreased PF count in adults, suggesting a potential link between PF number and OS levels in the ovaries (Lim et al., 2015). Additional research is required to understand how fluctuating levels of ROS occurring within PFs influence oocyte development. Excessive ROS was recently shown to enhance lipid peroxidation, a potential triggering mechanism of an iron‐dependent cell death mechanism termed ferroptosis that is mechanistically distinct from apoptosis (Stockwell, 2022). Mechanisms regulating ferroptosis under physiological conditions are not fully understood, but both genetic and chemotherapy‐derived disruptions to redox balance have been shown to induce ferroptosis in oocytes, leading to reduced oocyte quality, diminished ovarian reserves, and POI (Hu et al., 2021; Wang, Liu et al., 2022 Wang, Zhang et al., 2022; Zhang et al., 2023).

Here, we examine the impact of ROS on the dormancy of PFs and determine how oocytes within PFs respond to increased ROS or reduced antioxidant defenses, stimuli that jointly promote cell death. We find that mouse PFs are able to assume a prolonged state of dormancy through expressing high levels of antioxidant enzymes, preventing ROS‐mediated damage. With advancing age, ROS accumulation within ovaries is a likely driver of induced ferroptosis within nongrowing oocytes (NGOs). Finally, greater SOD1 expression within NGOs contributes to the maintenance of PF dormancy by inhibiting ROS.

## 2 RESULTS

### 2.1 ROS regulates the maintenance of primordial follicular dormancy

**FIGURE 1:**
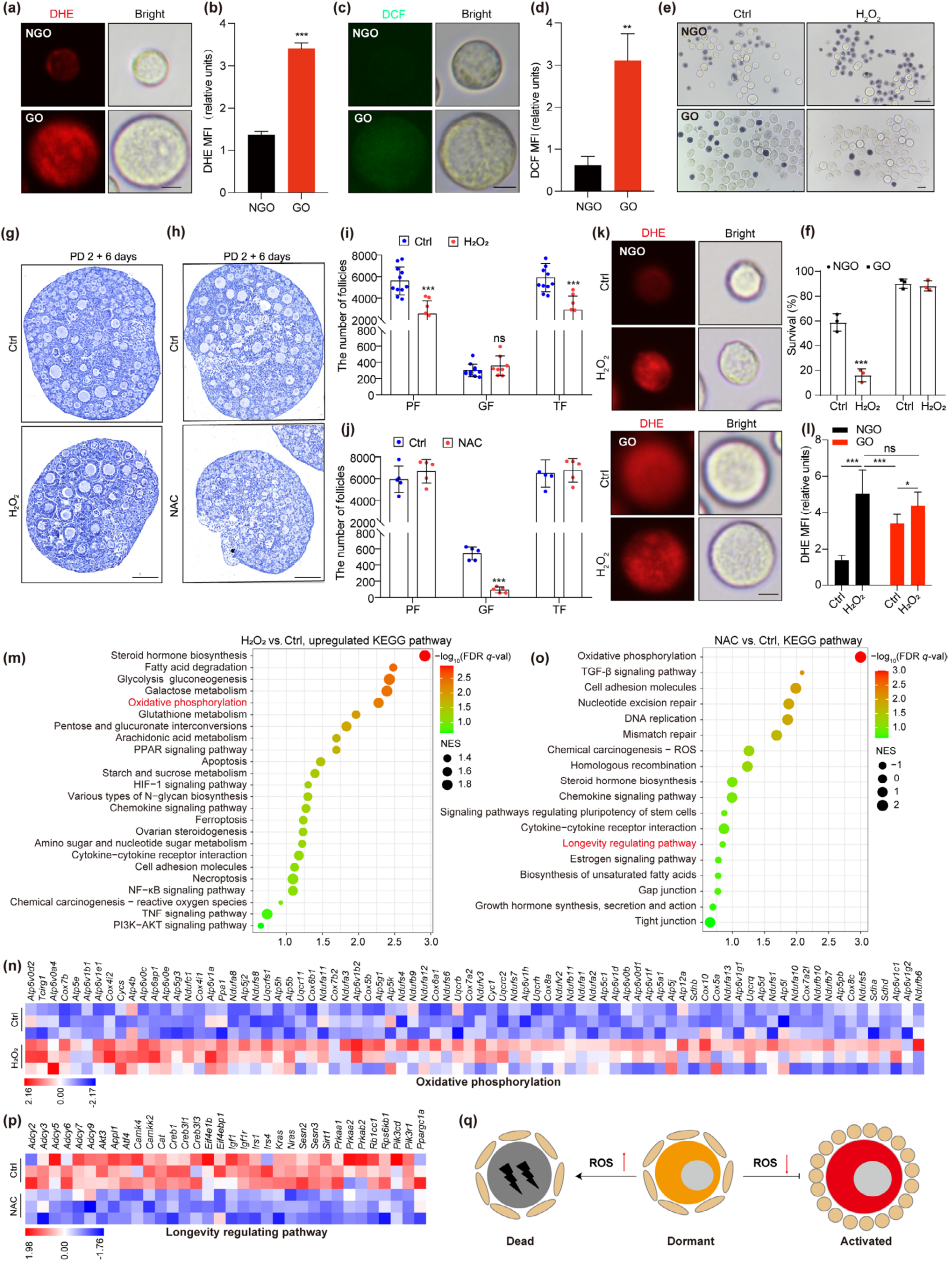
Effects of ROS levels on the PF maintenance. (a, c) Live‐cell imaging of NGOs (<20 μm) and GOs (>30 μm). The ROS levels were measured respectively using DHE (a) and DCF fluorescent probes (c). Scale bar, 10 μm. (b, d) Quantification of the mean fluorescence intensity (MFI) of DHE (b) and DCF (d) in oocytes (*n* = 3). (e, f) The survival rate of NGOs and GOs after 4 days of treatment with 1 mM H_2_O_2_ or not measured by Trypan blue staining (*n* = 3). (g, h) Representative images of sections from PD 2 (PFs have formed) ovaries cultured for 6 days with treatment of 1 mM H_2_O_2_ (g) and 10 mM NAC (h) or not. Scale bar, 100 μm. (i, j) Numbers of follicles at different developmental stages were counted in each ovarian after treatment with H_2_O_2_ (*n* = 8) (h) and NAC (*n* = 5) (i) or not. GF, growing follicle; TF, total follicle. (k) Representative images of DHE‐labeled ROS levels in NGOs and GOs from the ovary treated with H_2_O_2_ or not. Scale bar, 10 μm. (l) Quantification of the DHE mean MFI in NGOs and GOs (*n* = 3). (m, o) KEGG pathways by GSEA enrichment analysis of all genes in ovaries after treatment with H_2_O_2_ (m) and NAC (o) compared with those from Ctrl group respectively. *p* value < 0.05, FDR <0.25. (n) Heatmap illustration shows upregulated genes in oxidative phosphorylation pathway after treatment with H_2_O_2_. (p) Heatmap illustration shows downregulated genes in longevity regulating pathway after treatment with NAC. (q) Mode pattern shows the effect of ROS levels on the PF maintenance. ***p < 0.001, **p < 0.01, *p < 0.05, ns, not significant.

To investigate ROS levels in PFs, NGOs of PFs and oocytes from growing follicles (GOs) were isolated based on their diameters. Dihydroethidium (DHE) and DCFH‐DA, which converts to DCF when oxidized, were used as probes to measure levels of intracellular ROS. The statistical results of the mean fluorescence intensity revealed that NGOs exhibited lower ROS levels compared to GOs (Figure 1a–d), indicating a potential role for ROS levels in the maintenance of PF dormancy. Subsequently, the isolated oocytes were subjected to an OS model of exposure to H_2_O_2_ for 4 days, and cell death was assayed via trypan blue staining. The results revealed that OS substantially increased NGO mortality but did not promote the death of GOs (Figure 1e,f).

To test how ROS influenced PF maintenance, we utilized a mouse ovarian in vitro culture system and two experimental protocols. The first protocol aimed to induce OS through H_2_O_2_ exposure, while the second protocol utilized N‐Acetyl Cysteine (NAC) to inhibit ROS. Through histological examination and section analysis, we determined that the PF pool in the OS model forms normally (Figure S1o, p), but PF loss was accelerated under OS. Meanwhile, PF activation was notably suppressed by ROS inhibition (Figure 1g–j). Furthermore, in agreement with the observation that ROS levels are elevated in GOs compared to NGOs in vivo, OS increased ROS levels to a large extent in NGOs (*p* < 0.01) but only mildly within GOs (*p* < 0.05) (Figure 1k,l), compared to control treatments. Collectively, these findings indicate that OS predominantly harms PFs rather than growing follicles.

We performed whole‐transcriptome sequencing on mouse ovaries cultured in vitro to identify pathways altered by ROS affecting PF maintenance. We employed gene set enrichment analysis (GSEA), identifying a significant upregulation of multiple metabolic pathways in the OS model, including oxidative phosphorylation (Figure 1m,n). This suggests that altered metabolic profiles might have a functional role in the loss of PF maintaining under OS. Furthermore, GSEA revealed an enrichment of three cell death mechanisms in the OS model, including ferroptosis, apoptosis, and necroptosis (Figure 1m), suggesting that the potential loss of PFs might be occurring through these mechanisms. Additionally, within the ovarian culture model, we observed the substantial suppression of signaling pathways associated with ovarian development (Figure 1o) and regulating ovarian longevity following ROS inhibition (Figure 1p). This suggests that an optimal level of ROS is essential for PF maintenance and activation.

Taken together, these findings underscore the importance of balancing ROS levels for the homeostatic maintenance of PFs, as depicted in Figure 1q.

### 2.2 The dormancy of PFs depends on the higher levels of SOD1 present in NGOs

**FIGURE 2:**
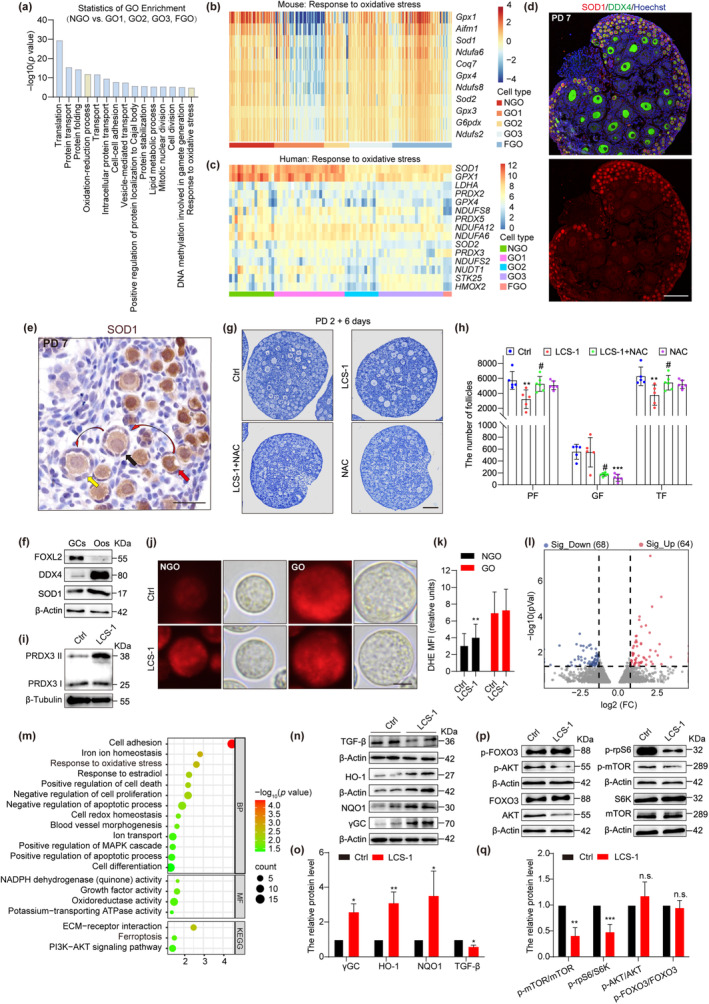
Ngo maintains dormancy depending on higher level of SOD1. (a) Gene ontology analysis of upregulated DEGs in NGOs. Original analysis data came from GSE114822. *p* value < 0.01, FDR <0.25. (b) Heatmap illustration shows response to oxidative stress pathway in mouse. (c) Heatmap illustration shows response to oxidative stress pathway in human. Original analysis data came from GSE107746. (d) Immunofluorescence staining for SOD1 (red). Oocytes were stained with DDX4 (green) and the nuclei were stained with hoechst (blue). Scale bar, 100 μm. (e) Immunohistochemistry staining for SOD1. The red arrow indicates the PFs in a dormant state, the black arrow indicates the PFs in an activated state and the yellow arrow indicates the primary follicle. Scale bar, 50 μm. (f) Immunoblotting of SOD1 in oocytes (Oos, indicated by DDX4) and granulosa cells (Gcs, indicated by FOXL2) of PD 5 mouse ovaries. (g) Representative images of PD 2 ovaries cultured for 6 days with treatment of 5 μM LCS‐1 (SOD1‐specific active inhibitor) and 10 mM NAC or not. Scale bar, 100 μm. (h) Numbers of follicles at different developmental stages in each ovaries (*n* = 5). * represents LSC‐1 group versus Ctrl group, # represents LSC‐1 + NAC group versus LCS‐1 group. (i) Nonreducing immunoblotting of PRDX3 in ovaries. Monomeric and dimeric PRDX3 are indicated. (j) Representative images of DHE‐labeled ROS levels detection in NGOs and GOs. Scale bar, 10 μm. (k) Quantification of the DHE MFI in NGOs and GOs (*n* = 3). (l) Volcano plot shows DEGs (upregulated, red; downregulated, blue) in LCS‐1 treatment ovaries compared to the Ctrl. *p* value < 0.05, Fold Change ≥2. (m) Gene ontology and KEGG analysis of DEGs in LCS‐1 treatment ovaries compared to the Ctrl. *p* value < 0.05, FDR <0.25. (n, o) Immunoblotting and the relative expression levels of TGF‐β, HO‐1, γGC, and NQO1 to β‐Actin. (p, q) Immunoblotting and the relative expression level of p‐mTOR/mTOR, p‐rpS6/S6K, p‐AKT/AKT, and p‐FOXO3/FOXO3 to β‐Actin. ***p < 0.001, **p < 0.01, *p < 0.05, ns, not significant, #*p* < 0.05.

To investigate the major causes of differing ROS levels between NGOs and GOs, gene expression patterns were examined using published single‐cell transcriptomic data (Gu et al., 2019). Next, gene ontology enrichment analysis was performed on genes that were found to be highly expressed in NGOs compared to GOs. The results indicated that genes enriched in NGOs are associated with oxidation–reduction processes as well as pathways responding to OS (Figure 2a). We used heat to display the expression of genes involved in OS response at different oocyte developmental stages. This analysis revealed that the expression of these genes is significantly reduced upon PF activation in both mice and humans (Figure 2b,c).

Among the differentially expressed genes (DEGs), we observed the antioxidant gene *Sod1*, with established critical roles in reproductive processes of female mice, to be expressed at greater levels in NGOs compared to GOs (Noda et al., 2012). By immunofluorescence and histochemical staining, we further determined that SOD1 is highly and specifically expressed in dormant PFs and becomes down‐regulated upon follicle activation (Figure 2d,e; Figure S1a). Furthermore, we isolated granulosa cells and oocytes from mouse ovaries and examined SOD1 expression, demonstrating the SOD1 expression levels in oocytes to be significantly greater than the level expressed by granulosa cells (Figure 2f, Figure S1k). We therefore hypothesized that SOD1 in NGOs might be essential for the maintenance of PF dormancy.

To test this hypothesis, we utilized LCS‐1, a specific inhibitor of SOD1, by adding it to cultured postnatal day (PD) 2 mouse ovaries that were maintained in vitro for 4 or 6 days. Examining sections of the cultured tissue revealed that although the inhibition of SOD1 did not affect PF formation after 4 days of culture (Figure S1h, i), PF loss occurred at a significantly greater rate, and SOD1 inhibition was associated with increased total follicle loss (Figure 2g,h). The conclusion was further supported by the addition of the ROS inhibitor NAC to the culture system, which rescued the loss of PFs following SOD1 inhibition (Figure 2g,h). Notably, LCS‐1 treatment did not significantly alter the number of growing follicles, implying that SOD1 inhibition might lead to the loss of PFs by increasing OS. We next examined the dimeric form of PRDX3, which is stimulated to form by oxidative stress, to indirectly evaluate ROS activity in the cultured ovaries (Rodríguez‐Nuevo et al., 2022). We found that SOD1 inhibition resulted in increased PRDX3 dimer formation, indicating a greater amount of environmental oxidative stress (Figure 2i; Figure S1j). Consistent with this observation, DHE staining further revealed increased ROS levels in NGOs following SOD1 inhibition. We also examined OS levels in GOs, which were not changed (Figure 2j,k), in agreement with our previous findings (Figure 1e,f).

To explore how SOD1 inhibition altered ovarian gene expression, whole‐transcriptomic sequencing was performed on ovaries which did or did not receive LCS‐1 treatment. After visualizing DEGs using a Volcano plot, we identified 64 upregulated and 68 downregulated genes in response to LCS‐1 treatment of ovaries (Figure 2l). Kyoto Encyclopedia of Genes and Genomes (KEGG) and gene ontology enrichment analyses identified DEGs in pathways essential for PF maintenance, including PI3K‐AKT signaling, Extracellular Matrix (ECM) receptor interactions, and MAPK signaling (Figure 2m). These pathways have previously been associated with POI (Shi et al., 2023). We validated these results by performing Western blotting to detect the anticipated downregulation of TGF‐β, which is associated with ECM receptor interactions in ovaries (Figure 2n,o). Collectively, these findings suggest a significant role for SOD1 in PF maintenance.

Previous investigations have suggested that one potential factor contributing to the loss of PFs is the overactivation of signaling pathways associated with stress, such as the mTOR signaling pathway in pregranulosa cells and the AKT‐FOXO3 pathway in oocytes (Castrillon et al., 2003; John et al., 2008; Li et al., 2010). To test if SOD1 inhibition led to PF overactivation, protein levels for components of the mTOR–AKT‐FOXO3 signaling pathway were examined following SOD1 inhibition. Western blotting revealed that while mTOR–AKT signaling was suppressed, FOXO3 levels and phosphorylation status were unchanged, suggesting SOD1 inhibition did not affect PF activation (Figure 2p,q). Alternatively, DEG analysis revealed an enrichment for genes in several pathways related to the oxidative stress response and other biological processes, including apoptosis, cell proliferation, ferroptosis, and iron ion homeostasis (Figure 2m). Western blotting results similarly confirmed a significant increase in proteins responding to OS in ovaries following SOD1 inhibition (Figure 2n,o). Therefore, SOD1 inhibition likely disrupts the antioxidant capacity of the ovarian culture system in vitro, affecting PF survival rather than PF activation.

Though we did not observe an effect of SOD1 inhibition on FOXO3 expression, a correlation between SOD1 and FOXO3 levels during oocyte activation was observed. Immunofluorescence staining results revealed that FOXO3 expression was initiated in oocytes during PF formation during embryonic days 16.5 to 18.5, which parallels the rise of SOD1 (Figure S1b). Similarly, both FOXO3 and SOD1 were less abundant in growing follicles compared to PFs in the ovaries of PD 7 mice. Moreover, decreased SOD1 levels were shown to be associated with a lack of nuclear FOXO3 expression (Figure S1c), indicating a coordinated expression pattern between the two proteins in PFs. To examine this relationship further, oocytes isolated from PD5 ovaries were used as an input to identify genome‐wide FOXO3 binding using Cut&Tag followed by sequencing (Figure S1d,e). The results revealed an enrichment for FOXO3 binding of genes related to the oxidative stress response, although FOXO3 did not appear to directly regulate *Sod1*. However, *Sod1* might be indirectly regulated through any of the 15 genes bound by FOXO3 that respond to OS (Figure S1f,g). Collectively, these findings imply that FOXO3 has an active role in modulating *Sod1* expression to sustain PF dormancy. Together, these data suggest that PF dormancy is sustained by maintaining high levels of SOD1 in NGOs that counteract moderate levels of ROS.

### 2.3 The maintenance of PFs by SOD1 is correlated with age

**FIGURE 3:**
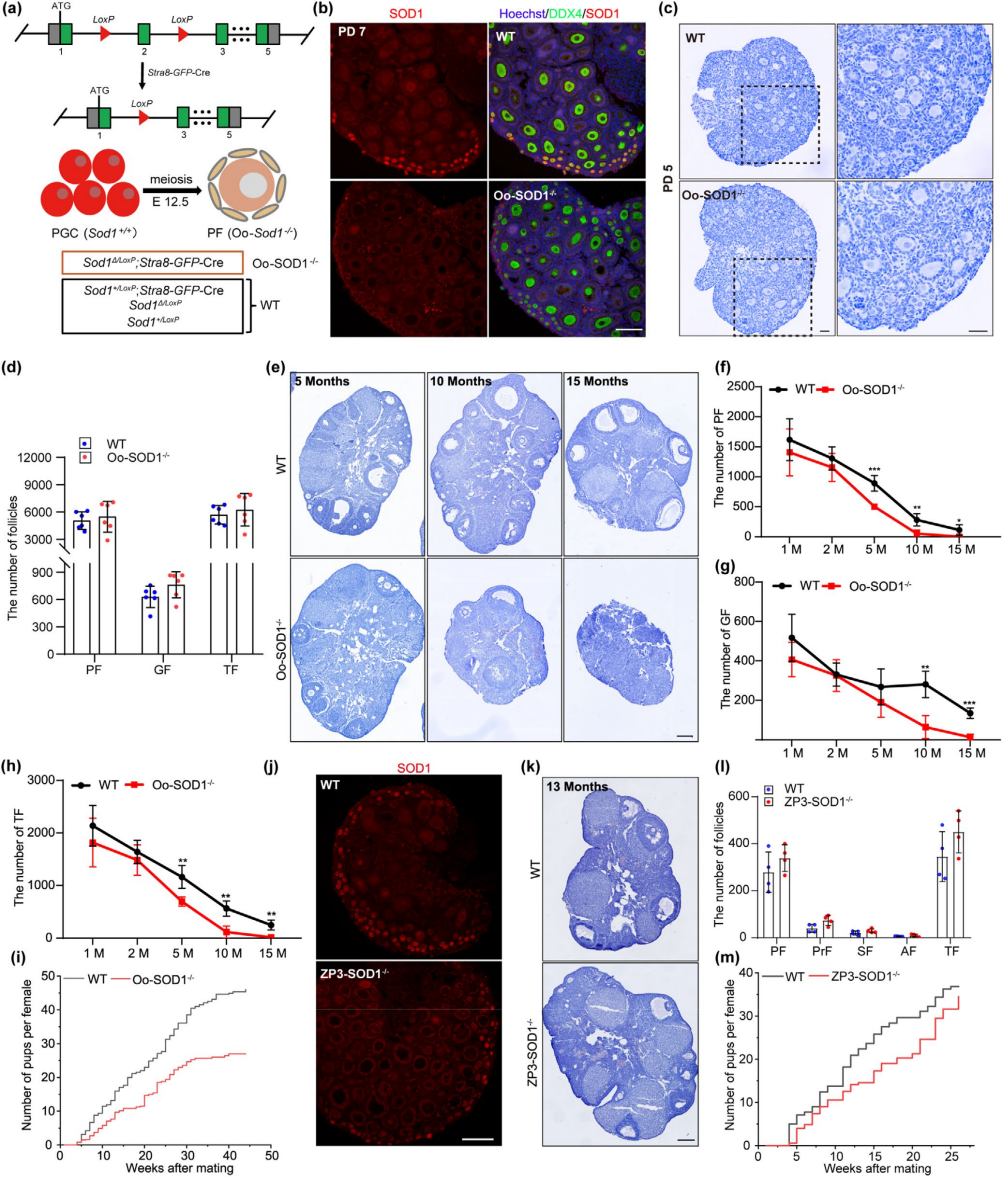
SOD1 shows an age‐dependent effect on supporting PF maintenance. (a) A scheme for mouse model of Oo‐SOD1^−/−^. *Stra8‐GFP* drives the expression of Cre enzyme during the meiosis initiation phase of oocytes, resulting in recombinant of the *LoxP* site on *Sod1‐2* exon. ATG, translation start codon; PGC, primordial germ cells. (b) Immunofluorescence staining for SOD1 (red) was used to detect knockout efficiency in PD 7 oocytes. Oocytes were stained with DDX4 (green) and the nuclei were stained with hoechst (blue). Scale bar, 100 μm. (c) Representative images of ovarian sections from PD 5 WT and Oo‐SOD1^−/−^ mice (PF pool was established). Scale bar, 50 μm. (d) Numbers of follicles at different developmental stages were counted in each ovary from WT and Oo‐SOD1^−/−^ mice (*n* = 6). There was no significant difference between the groups. (e) Representative images of ovarian sections from WT and Oo‐SOD1^−/−^ mice at different ages. Scale bars, 200 μm. (f, g, h) Numbers of PF (f), GF (g) and TF (h) were counted in each ovary from WT and Oo‐SOD1^−/−^ mice at different ages (*n* = 5). (i) Fertility testing of WT and Oo‐SOD1^−/−^ female mice (*n* = 20). (j) Immunofluorescence staining for SOD1 was used to detect knockout efficiency of growing oocytes by *Zp3*‐Cre in PD 7 ovaries. Scale bar, 100 μm. (k) Representative images of ovarian sections from WT and ZP3‐SOD1^−/−^ mice at 13‐month old. Scale bars, 200 μm. (l) Numbers of follicles at different developmental stages were counted in each ovary from WT and ZP3‐SOD1^−/−^ mice (*n* = 4). There was no significant difference between the groups. PrF, primary follicle, SF, secondary follicle, AF, antral follicle. (m) Fertility testing of WT and ZP3‐SOD1^−/−^ female mice (*n* = 8). ***p < 0.001, **p < 0.01, *p < 0.05.

To assess the physiological role of SOD1 in PF maintenance, we constructed genetically modified mouse models that targeted SOD1 in oocytes at different developmental stages. SOD1 was ablated in early meiotic stage oocytes by using *Stra8‐GFP*‐Cre (Chen et al., 2018; Lin et al., 2017). Mice with genotypes *Sod1* ^*LoxP/+*^; *Stra8‐GFP*‐Cre, *Sod1* ^*Δ/LoxP*^ and *Sod1* ^*LoxP/+*^ were classified as wild type (WT), while those with *Sod1* ^*Δ/Loxp*^; *Stra8‐GFP*‐Cre were designated as Oo‐SOD1^−/−^ KO mice (Figure 3a). Immunofluorescence confirmed the specific inactivation of *Sod1* in oocytes and not in somatic cells, as compared to WT controls (Figure 3b). We examined the PF pool at PD5, revealing similar numbers of PFs in WT and Oo‐SOD1^−/−^ mice, indicating that oocyte specific knockout of *Sod1* did not affect PF pool establishment (Figure 3c,d).

We next examined the influence of *Sod1* deletion on mouse fertility across a 15‐month age span. The resulting tissue sections and statistical analyses of the quantifications are presented in Figure 3e and Figure S2a–e, respectively. In WT mice ovaries, the number of PFs gradually declined with increasing age, and was completely depleted by 15 months (Figure 3f). In contrast, the number of growing follicles in WT ovaries was relatively stable from 2 to 10 months of age, and after which declined with age. By 15 months, only about 150 growing follicles remained in the ovaries. These findings indicate that in vivo, a time dependent change in the number of follicles in WT mice acts as a profound indicator of reproductive aging (Figure 3g). In Oo‐SOD1^−/−^ mice, total follicle numbers were similar to WT mice prior to 2 months of age. However, the rate of PF loss was increased and the number of PFs was reduced to half of WT levels mice by 5 months (Figure 3f). During the time, the number of growing follicles remained similar to that of WT mice (Figure 3g). Remarkably, PFs were almost completely depleted in Oo‐SOD1^−/−^ mice at 10 months, which resembled 15 months old WT ovaries (Figure 3f), and PF depletion was accompanied by a reduction in the number of growing follicles (Figure 3g). Finally, we not only observed almost a complete loss of available follicles in Oo‐SOD1^−/−^ mice at 15 months old (Figure 3h), but also a concurrent, highly significant decline in female fertility (Figure 3i). Overall, oocyte specific deletion of *Sod1* increased the rate of follicle loss and predisposed mice to infertility.

To determine if SOD1 also had important functions in growing follicles, we inactivated *Sod1* specifically in GOs by *Zp3*‐Cre (Figure 3j). Both the number of follicles in the ovaries and the female fecundity of ZP3‐SOD1^−/−^ mice were normal till 13 months of age. These results indicated that deletion of *Sod1* in GOs does not affect female reproduction (Figure 3k–m) and that the role of SOD1 in the oocytes must therefore be primarily involve the maintenance of the PF pool.

### 2.4 SOD1 maintains PF dormancy through antioxidant activity during aging in mice

**FIGURE 4:**
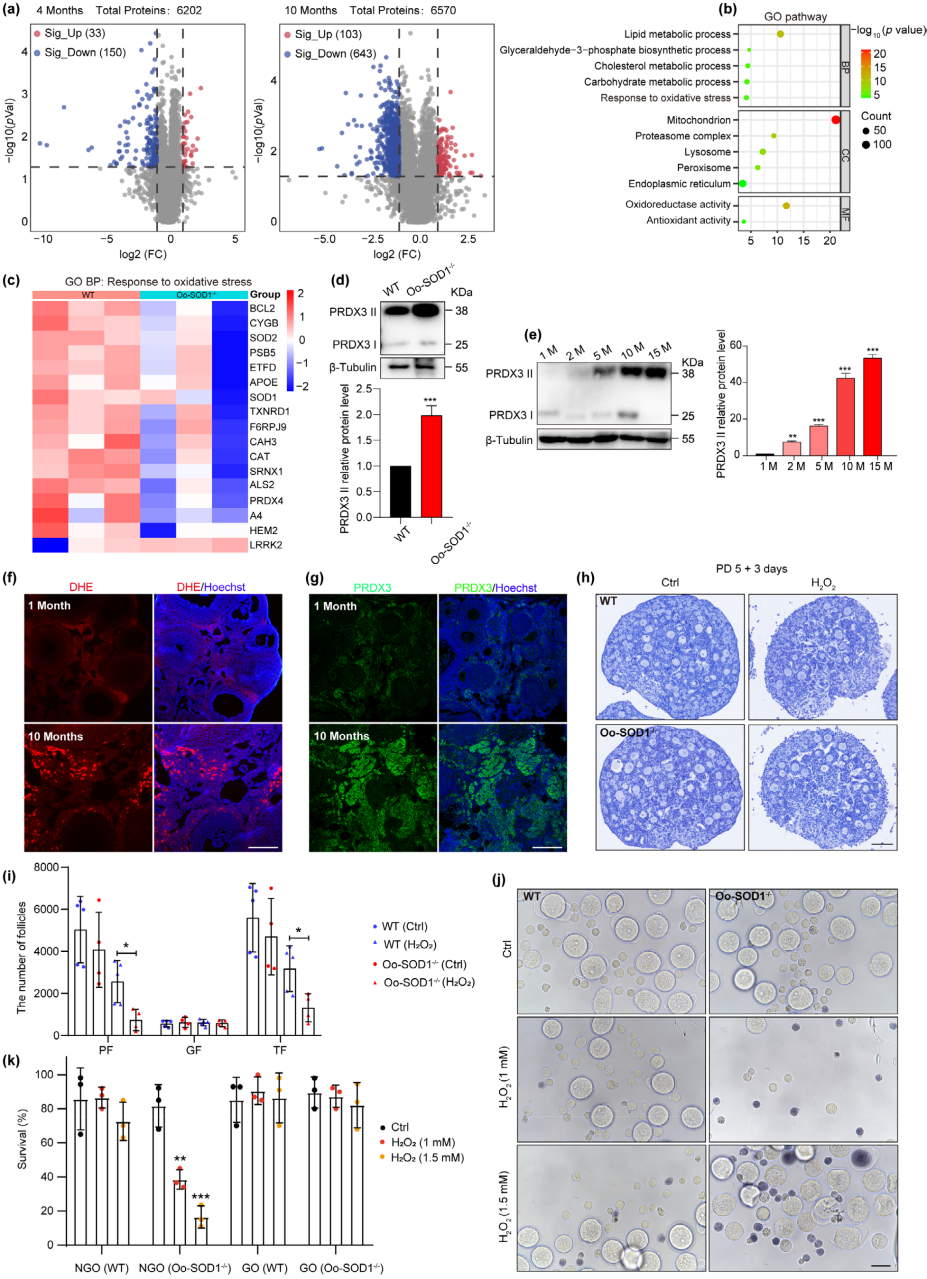
SOD1 maintains PF dormancy through an antioxidant effect during aging in mice. (a) Volcano plot shows differential expression proteins (upregulated, red; downregulated, blue) in ovaries from Oo‐SOD1^−/−^ mice compared to WT mice at the age of 4‐month‐old and 10‐month‐old. *p* value < 0.05, Fold Change ≥2. (b) Gene ontology analysis of differential expression proteins in ovaries of Oo‐SOD1^−/−^ mice compared to WT mice at the age of 10 months. *p* value < 0.05, FDR <0.25. (c) Heatmap illustration shows response to oxidative stress pathway in (b). (d) Immunoblotting of PRDX3 and relative expression level to β‐Tubulin in ovaries of WT and Oo‐SOD1^−/−^ mice at the age of 10 months. (e) Immunoblotting of PRDX3 and relative expression level to β‐Tubulin in ovaries in mice of different ages. (f) DHE labeling to detect ROS levels in the ovaries of 1‐month old and 10‐month‐old mice. The nuclei were stained with hoechst (blue). Scale bars: 100 μm. (g) Immunofluorescence staining for PRDX3 was used to detect ROS levels in ovaries at the age of 1 month and 10 months. The nuclei were stained with hoechst (blue). Scale bars: 100 μm. (h) Representative images of sections from WT and Oo‐SOD1^−/−^ PD 5 ovaries cultured for 3 days with treatment of 1 mM H_2_O_2_ or not, respectively. Scale bar, 100 μm. (i) Numbers of follicles at different developmental stages were counted in each ovary (*n* = 4). (j, k) Representative images of ovarian sections from WT and Oo‐SOD1^−/−^ mice on PD 5, cultured for 3 days with or without treatment of 1 mM H_2_O_2_. Scale bar, 50 μm. ***p < 0.001, **p < 0.01, *p < 0.05.

To investigate the impact of inactivating *Sod1* on ovarian function at different ages, proteomic analysis was conducted on ovaries isolated from 4‐ and 10‐month‐old WT and Oo‐SOD1^−/−^ mice. Volcano plots revealed 33 upregulated and 150 downregulated proteins in 4‐month‐old knockout ovaries, while the number increased to 103 upregulated and 643 downregulated proteins in 10‐month‐old mice (Figure 4a). This suggests that the physiological role of SOD1 in female reproduction is age dependent. Gene ontology enrichment analysis of differentially abundant proteins in 10‐month‐old ovaries further highlighted pathways linked to oxidative stress response, antioxidant activity, and various metabolic processes (Figure 4b). A heat map of 17 OS‐related proteins decreased in Oo‐SOD1^−/−^ ovaries further suggests OS is increased the absence of SOD1 (Figure 4c). Western blot analysis of PDRX3 dimerization status also supported increased OS in the absence of SOD1 at 10 months (Figure 4d).

Aging in mice was associated with progressive ROS accumulation within ovaries, as indicated by the gradual increase in PRDX3 homodimerization observed from 1 to 15 months by Western blot analysis (Figure 4e). DHE staining (Figure 4f) and PRDX3 immunofluorescence staining (Figure 4g) in mice aged 1 and 10 months suggested that physiological ROS could be produced by ovarian stromal cells, potentially providing a link to increased fibrotic remodeling occurring within the ovary during senescence. OS within SOD1 deficient resulted in increased levels of ROS‐induced cell death. We validated this observation in vitro by culturing ovaries from WT and Oo‐SOD1^−/−^ PD5 mice under an OS model for 3 days, which resulted in the marked reduction of PFs in knockout ovaries compared to the controls (Figure 4h,i). Subsequent isolation and culture of NGOs and GOs from both genotypes under OS conditions for 48 h also demonstrated that SOD1‐deficient NGOs are more prone to oxidative damage (Figure 4j,k). Together, these data support the pivotal role of SOD1 in modulating ovarian ROS homeostasis and cell viability.

### 2.5 Ferroptosis may be the main mechanism leading to the loss of PFs in mice

Inhibiting SOD1 in vitro resulted in increased expression of genes related to apoptosis and ferroptosis in mouse ovaries. To determine which cell death mechanisms were active within PFs following SOD1 inhibition, we inhibited apoptosis by Z‐VAD‐FMK in ovarian cultures. Notably, inhibiting apoptosis did not mitigate PF loss caused by SOD1 inhibition. We also did not observe changes in proteins associated with apoptosis and autophagy following SOD1 inhibition (Figure S3a–e). These findings suggest an alternative cell death mechanism is primarily responsible for the loss of PFs following SOD1 inhibition.

**FIGURE 5:**
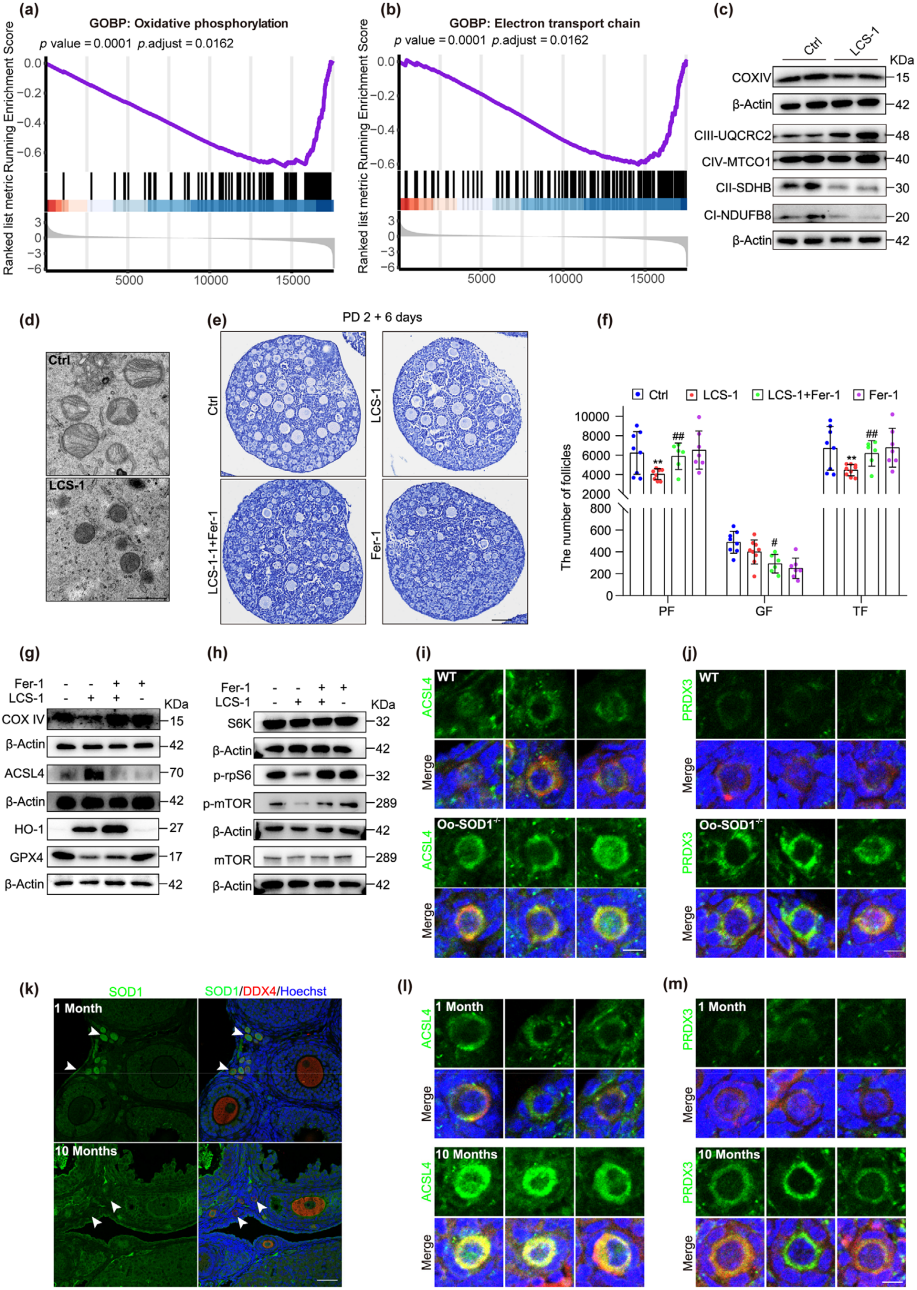
SOD1 inhibits PF ferroptosis through antioxidant effects during aging in mice. (a, b) GOBP: Oxidative phosphorylation pathway (a) and electron transport chain pathway (b) of GSEA enrichment analysis of all genes in LCS‐1 treatment ovaries compared to the Ctrl. *p* value = 0.0001, FDR = 0.0162. (c) Immunoblotting of proteins associated with the oxidative phosphorylation pathway. (d) The mitochondria in ovaries under the electron microscope. Scale bars, 1 μm. (e) Representative images of sections from PD 2 ovaries cultured for 6 days with treatment of LCS‐1 and 20 μM Ferrostatin‐1 (Fer‐1, selective ferroptosis inhibitors) or not respectively. Scale bar, 100 μm. (f) Numbers of follicles at different developmental stages were counted in each ovary (*n* = 6). * represents LSC‐1 group versus Ctrl group, # represents LSC‐1 + Fer‐1 group vs. LCS‐1 group. (g, h) Immunoblotting of proteins associated with the ferroptosis (g) and mTOR pathway (h) in ovaries treated with LCS‐1 and Fer‐1 or not. (i, j) Immunofluorescence staining for ACSL4 (i) and PRDX3 (j) of PFs in WT and Oo‐SOD1^−/−^ mice. Oocytes were stained with DDX4 (red) and the nuclei were stained with hoechst (blue). Scale bar, 10 μm. (k) Immunofluorescence staining for SOD1 in mouse ovarian sections at the age of 1 month and 10 months. The oocyte was stained with DDX4 (red) and the nucleus was stained with hoechst (blue). The white arrow indicates the PF. Scale bar, 50 μm. (l, m) Immunofluorescence staining for ACSL4 (l) and PRDX3 (m) of PFs in mice at the age of 1 month and 10 months. Oocyte were stained with DDX4 (red) and the nuclei were stained with hoechst (blue). Scale bar, 10 μm. **p < 0.01, #*p* < 0.05, ##*p* < 0.01.

These results, in combination with the increase in expression of ferroptosis‐related genes, suggested that ferroptosis might be the principal mechanism underlying PF demise following SOD1 inhibition. To determine if PF cell death occurred via ferroptosis, we leveraged bulk RNA‐sequencing data obtained from cultured ovarian tissues with and without treatment with the SOD1 inhibitor LCS‐1. GSEA revealed the significant downregulation of the oxidative phosphorylation and electron transport chain pathways, which are essential for mitochondrial function (Figure 5a,b). This suggested that SOD1 inhibition results in mitochondrial dysfunction in ovarian tissues. We further observed the downregulation of proteins associated with multiple mitochondrial complexes, corroborated these findings (Figure 5c, Figure S3f). Additionally, mitochondrial morphology revealed by electron microscopy showed decreased mitochondrial volumes and cristae densities following SOD1 inhibition. These changes are consistent with morphological and functional changes observed in mitochondria during ferroptosis (Figure 5d).

To determine if ferroptosis might be responsible for the loss of PFs induced by SOD1 inhibition, we employed an in vitro ovarian culture model with inhibition of both SOD1 and ferroptosis, using ferroptosis inhibitor Fer‐1. The inhibition of ferroptosis significantly mitigated PF loss, as shown by ovarian histology and follicle count analysis (Figure 5e,f). ACSL4 stimulates ferroptosis by promoting long‐chain polyunsaturated fatty acid synthesis, leading to increased levels of lipid peroxidation. Conversely, GPX4, a key antioxidant enzyme, prevents ferroptosis by reducing lipid peroxidation levels (Doll et al., 2017; Ingold et al., 2018). Inhibiting SOD1 resulted in increased ACSL4 levels and decreased GPX4 levels. Fer‐1 treatment was shown to suppress ACSL4 upregulation and GPX4 downregulation. Additionally, Fer‐1 restored the protein levels of the mitochondrial marker COXIV. Fer‐1 inhibition also resulted in increased levels of HO‐1, indicating an enhanced response to OS, as previously observed in glioma cells (Ling et al., 2022) (Figure 5g, Figure S3g–j). We observed an increase in mTOR signaling upon Fer‐1 treatment, suggesting this molecular mechanism alleviates PF loss induced by SOD1 inhibition when ferroptosis is inhibited (Figure 5h, Figure S3k,l). Examining the expression of ACSL4 and PRDX3 in PFs of WT and Oo‐SOD1^−/−^ mice revealed that both proteins were significantly increased, confirming that *Sod1* knockout in oocytes triggers ferroptosis in PFs (Figure 5i,j, Figure S3m,n).

These results reveal that ferroptosis as the predominant cause of NGO death in both the Oo‐SOD1^−/−^ mouse model and the in vitro ovarian culture model with SOD1 inhibition, suggesting that OS in the oocyte stimulates ferroptosis. Given that ovarian aging is characterized by ROS accumulation, we speculated that ferroptosis may be the principal pathway leading to PF loss during ovarian aging. To validate this hypothesis, we performed immunofluorescence staining of several key proteins, demonstrating that at 10 months of age SOD1 is downregulated in PFs compared to 1‐month‐old mice (Figure 5k), while ACSL4 expression is upregulated (Figure 5l, Figure S3o). Additionally, PRDX3 was significantly upregulated in 10‐month‐old PFs. Notably, this study revealed PRDX3 as an oxidative marker indicative of ferroptosis (Figure 5m, Figure S3p), which is in consistent with a recent report (Cui et al., 2023). Collectively, in dormant PFs of aging adults, we show that ROS accumulation due to the downregulation of antioxidant molecules leads to oocyte ferroptosis, which may be the primary mechanism underlying the loss of PFs and the progression of female reproductive senescence.

## 3 DISCUSSION

This study provides substantial evidence linking ROS levels in NGOs to the fate determination of PFs. The levels of ROS in oocytes were shown to depend on their developmental stage. For instance, early‐stage oocytes in humans and *Xenopus* exhibit minimal ROS, potentially due to the inactivation of mitochondrial complex I (Rodríguez‐Nuevo et al., 2022). This study also confirms that mouse NGOs exhibit lower ROS levels, which increase in GOs of developing follicles. Interestingly, NGOs express genes associated with mitochondrial complex I, including NDUFB8, in all oocytes (Figure S1l,m). To uncover the mechanisms responsible for the ROS‐suppressing capacity in NGOs and GOs, single‐cell sequencing data revealed that NGOs develop a strengthened antioxidant capacity, characterized by elevated levels of GPX1 and SOD1 in oocytes (Figure 2d, Figure S1n). Notably, the heightened expression of SOD1 contributes significantly to the diminished ROS levels in NGOs. Collectively, this study underscores the pivotal role of SOD1 in maintaining oocyte health and female fertility, aligning with existing literature that highlights the importance of antioxidants in mitigating ROS‐induced reproductive decline (Matzuk et al., 1998; Perkins et al., 2019).

The functions of SOD1 in the ovaries are cell type‐specific, concentration‐dependent, and correlate with aging. When *Sod1* was selectively knockout in GOs or granulosa cells using *Zp3*‐Cre or *Foxl2*‐Cre, respectively, it did not significantly affect follicle counts nor induce reproductive aging (Figure 3k,l and S2h–k). This suggests SOD1 acts to prevent ovarian aging through cellular functions exerted specifically in NGOs. In contrast, examining the Oo‐SOD1^−/−^ mouse model revealed that SOD1 deletion reduced PF number by 5 months of age and resulted in reproductive senescence by 10 months. This phenotype agrees with the expected result of ROS accumulation and represents an accelerated loss of PFs compared to physiological ovarian aging mice. According to Faddy & Gosden, differential rates of PF loss, especially the increased rates observed after the age of 38 in humans, are correlated with menopause. If the accelerated rate of PF depletion could be mitigated, reproductive lifespan could potentially be lengthened to 71, rather than 51 (1995). High levels of SOD1 expression may have functional roles in the ovary beyond PF maintenance, as we observed GOs isolated from Oo‐SOD1^−/−^ mice exhibited complete degeneration after 7 days of in vitro culture (Figures S2f,g). Finally, SOD1 may also have a functional role in ovarian somatic cells, as we observed significantly diminished fertility of Gc‐SOD1^−/−^ mice despite unaffected PFs (Figure S2l). According to Noda et al., 2012, systematic SOD1 deletion in mice results in decrease progesterone levels and smaller corpus luteum size, in pregnant mice or mice primed with gonadotropin for 30 h. This could be associated with impaired progesterone secretion by the corpus luteum (2012). However, further studies are needed to confirm this hypothesis.

As the ovaries aged, we observed ROS accumulation resulting in altered cellular metabolism and the induction of ferroptosis in oocytes. Ferroptosis, a form of cell death, is linked to an imbalance between ROS production and antioxidant defenses (Yang & Stockwell, 2016). With increasing age, levels of toxic lipid peroxides found within NGOs increases, resulting in damage to cell membranes and ferroptosis (Bock & Tait, 2020; de Bruin et al., 2002; Faddy & Gosden, 1995). Here, we show that inhibiting ferroptosis could prevent PF loss in SOD1‐deficient oocytes. Additionally, both *Sod1* inactivation or advanced biological ages promoted ROS accumulation and induced significantly increased levels of ACSL4 and PRDX3, underscoring the role of ferroptosis in PF loss and the protective function of SOD1. Ferroptosis involves metabolic pathways like amino acids, iron, and polyunsaturated fatty acids (Stockwell et al., 2017), and its involvement is therefore further supported by our in vitro findings of enriched metabolic pathway abnormalities in mouse ovarian OS models. OS is a key factor driving senescence of the female reproductive system, with oocytes being notably sensitive to ROS (Aitken, 2020; Jeelani et al., 2018; Lord et al., 2015). Thus, the OS caused by SOD1 deletion in oocytes initiates ferroptosis, ultimately resulting in PF loss.

The interplay between ROS and ovarian health has driven interest in examining antioxidants for the ability to preserve PFs and delay reproductive senescence. Despite their promise, clinical applications have had mixed results, further underscoring the complexity of ROS‐antioxidant dynamics in the ovary (Chandel & Tuveson, 2014). Our study highlights the dual role of ROS in female aging, showing that while excessive ROS levels degrade PFs and impair oocyte mitochondrial function, moderate levels of ROS are required to maintain regular physiological processes and to prevent disease by modulating key signaling pathways including NFκB, MAPK, p53, and KEAP1‐NRF2 (Finkel & Holbrook, 2000). Until now, the positive effects of ROS on PF development had not been well‐defined. Here we show that inhibition of ROS significantly alters critical signaling pathways and suppresses PF activation. Together, these results suggest that a nuanced, balanced approach to ROS management is crucial for optimizing reproductive health and extending the reproductive lifespan.

**FIGURE 6:**
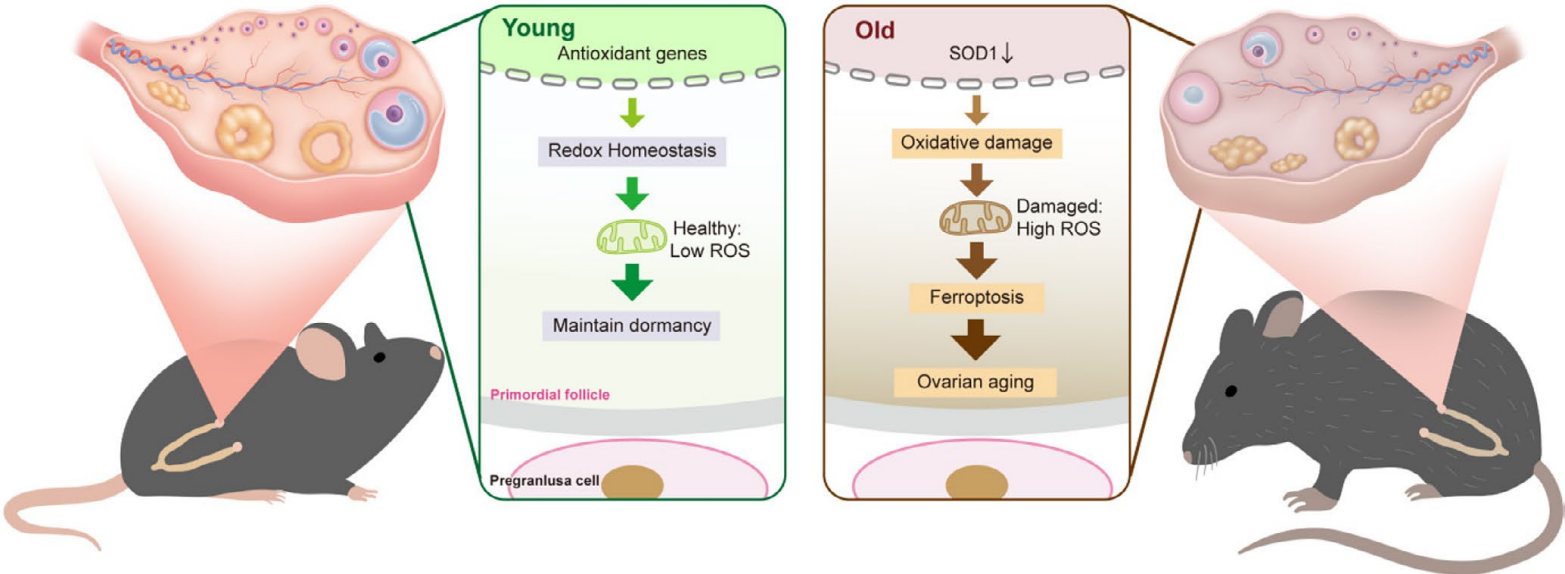
SOD1 inhibits ferroptosis of the PF by antioxidant effect and maintains PF dormancy. NGO of young mice exhibit higher expression of antioxidant enzymes, maintaining oocyte ROS at lower levels. With increasing age, the expression of SOD1 in NGO declines, leading to ROS accumulation, triggering primordial follicle loss due to ferroptosis, subsequently resulting in ovarian aging.

In summary, the balance between ROS and the antioxidant system in NGOs is critical for maintaining PFs and preserving fertility. Diminished SOD1 levels within NGOs of aging mice lessens their protection against OS, resulting in oocyte ferroptosis and the physiological decline of PFs, as illustrated in Figure 6. These insights provide a theoretical foundation for a deeper understanding of reproductive aging and the potential therapeutic application of antioxidants in the prevention and treatment of POI.

## 4 EXPERIMENTAL PROCEDURES

### 4.1 Mice feeding and treatments

ICR and C57BL/6 mice were procured from Beijing Vital River Experimental Animals Center (Beijing, China). *Sod1* ^*LoxP/LoxP*^ mice were generated by Gempharmatech (Jiangsu, China) using CRISPR–Cas9 technology. *Stra8‐GFP‐*Cre mice were generated by Prof. Minghan Tong (Shanghai Institute of Biochemistry and Cell Biology, Chinese Academy of Sciences, Shanghai, China). *Zp3‐*Cre mice were generated by Prof. Fengchao Wang (Transgenic Animal Center, National Institute of Biological Sciences, Beijing, China), and *Foxl2*‐Cre mice were generated by Prof. Fei Gao (Institute of Zoology, Chinese Academy of Sciences, Beijing, China). Mice were housed under a 12‐h light–dark cycle, at a room temperature of 20–25 °C, humidity of 55% ± 10% and with free access to food and water. The animal experiments conformed to the guidelines and regulatory standards of the Institutional Animal Care and Use Committee of China Agricultural University, No. AW81601202‐3‐8.

Oo‐SOD1^−/−^ mice, ZP3‐SOD1^−/−^ mice and Gc‐SOD1^−/−^ mice were generated by mating *Sod1* ^*LoxP/LoxP*^ females with *Sod1* ^*LoxP/+*^; *Stra8‐GFP*‐Cre, *Sod1* ^*LoxP/LoxP*^; *Zp3*‐Cre and *Sod1* ^*LoxP/LoxP*^; *Foxl2*‐Cre males, respectively. For the breeding studies, both WT and gene‐edited female mice (Oo‐SOD1^−/−^, ZP3‐SOD1^−/−^, or Gc‐SOD1^−/−^) were housed individually with male C57BL/6 mice of established fertility. The cohabitation was monitored to record the litter size and reproductive output. Fetal and neonatal mice were derived from the mating of ICR male and female mice. In these mating experiments, two female mice were paired with one male mouse. The presence of vaginal plugs was used to designate embryonic day (E) 0.5, and the day of birth was designated as postnatal day (PD) 1.

### 4.2 Antibodies

A panel of antibodies was sourced from various reputable suppliers for use in our experiments. The rabbit monoclonal anti‐AKT (pan) and anti‐p‐AKT (S473), along with rabbit polyclonal antibodies against FOXO3, p‐FOXO3, TGFβ, p‐mTOR, mTOR, S6K, p‐rpS6, c‐Caspase3, and LC3 were all procured from Cell Signaling Technology. The rabbit polyclonal anti‐GPX1 antibody was acquired from Thermo Fisher Scientific, while the mouse monoclonal anti‐SOD1 and anti‐γGCSc antibodies were obtained from Santa Cruz Biotechnology. A suite of antibodies including rabbit polyclonal anti‐NQO1 and mouse monoclonal anti‐β‐Actin and anti‐β‐Tubulin were sourced from Abmart. Further, rabbit monoclonal anti‐HO‐1, anti‐SOD1, anti‐GPX4, anti‐FACL4/ACSL4, and anti‐DDX4 antibodies were purchased from Abcam. Additionally, the mouse monoclonal anti‐DDX4, mouse polyclonal anti‐OXPHOS, goat polyclonal anti‐FOXL2, and rabbit polyclonal anti‐COX4‐I1 antibodies were also acquired from Abcam. The rabbit polyclonal anti‐NDUFB8 and anti‐PRDX3 antibodies were obtained from NOVUSBIO, while Boster Biological Technology supplied the rabbit polyclonal anti‐NDUFB8 and anti‐PRDX3 antibodies.

### 4.3 Ovarian culture in vitro and follicles count

Ovaries from PD 2 ICR mice and PD 5 C57/BL6 mice were cultured in suspension on 6‐well plates (Corning) with 1.2 mL of DMEM/F12 culture medium (Gibco) supplemented with 1% penicillin–streptomycin 100 × solution (HyClone), and placed in an incubator at 37°C with 5% CO_2_ as reported by Zhang et al., 2024. Ovaries were treated with 1 mM H_2_O_2_, 10 mM NAC (Sigma‐Aldrich), 5 μM LCS‐1 (MCE), 20 μM Fer‐1 (MCE) or 10 μM Z‐VAD (MCE) for 3–6 days with media changed every 2 days. After the completion of the culture, ovaries were fixed in PBS solution containing 4% polyformaldehyde, embedded in paraffin embedding after alcohol gradient dehydration, cut into 5 μm thickness continuous sections and finally stained with H&E.

Primordial follicles (PFs) and primary follicles (PrFs) were counted in every fifth section (25 μm interval), and in each section, only those follicles with a clear oocyte nucleus were counted and multiplied by 5 to calculate the total number of follicles in an ovary. Secondary and antral follicles were counted in every section. Counting should be based on clear observation of the maximum boundary of the oocyte nucleus as the standard for follicles, and observe both anterior and posterior boundaries to avoid duplicate counting. Ovarian follicles at different developmental stages were distinguished from each other by standards established by Peterson and Peters (Pedersen & Peters, 1968).

### 4.4 Oocyte isolation and in vitro culture

Ovaries from PD 9 mice or cultured in vitro were digested in DMEM containing 0.25% trypsin (Gibco) for 20 min at 37 °C with occasional swirling. Individual cells were separated and trypsin was neutralized by adding 10% FBS (Gibco). Subsequently, oocytes were picked under a dissecting microscope, and categorized based on their diameter, specifically grouping those with a diameter of 20 μm or less as primordial follicle oocytes (nongrowing oocytes, NGOs) and those exceeding 20 μm as growing follicle oocytes (GOs). All oocyte imaging experiments were conducted in DMEM/F12 medium (Gibco) containing 10% FBS at 37 °C in a 5% CO_2_ incubator, and a thin layer of mineral oil (Sigma‐Aldrich) covered the medium on 4‐well plates (Thermo Fisher Scientific). In the oxidative stress model, trypan blue (Solarbio) was added to the medium with the aim of labeling the dead cells.

### 4.5 Oocyte ROS levels monitoring

For measurement of the ROS content in oocytes, ovaries were digested into individual cells and incubated in the corresponding solution containing 5 μM DHE (Sigma‐Aldrich) or 1× DCFH‐Da (Beyotime) at 37°C for 30 min. The cells were resuspended with DMEM/F12 medium containing 10% FBS after the fluorescence probe were removed by centrifuge, and then NGOs and GOs were picked under a dissecting microscope (Nikon) to capture the oocyte images. The mean fluorescence intensity was calculated using Adobe Photoshop software.

### 4.6 Ovary ROS levels monitoring by DHE

For measurement of the ROS levels in ovary, 1‐month‐old and 10‐month‐old mice were intraperitoneally injected with 20 mg/kg DHE, and repeated 30 min later. Ovaries were collected after 18 h, embedded in OTC after dehydration by 30% sucrose solution for frozen sectioning. Sections images were captured and nuclei were labeled by hoechst (Beyotime).

### 4.7 Western blotting

Reductive denaturing SDS‐PAGE was performed for common proteins detection. For PRDX3 analysis, ovaries were homogenized by crushing in Pierce™ IP Lysis Buffer (Thermon) containing 100 mM N‐Ethylmaleimide (NEM, Sigma‐Aldrich). Nonreducing SDS protein loading buffer was used for SDS‐PAGE, and thermal denaturation was omitted after pyrolysis (Cox et al., 2010; Rodríguez‐Nuevo et al., 2022). After SDS‐PAGE, separated proteins were transferred to a PVDF transfer membrane (Millipore), and the membranes were incubated with specific primary and secondary antibodies sequentially. Protein bands were detected by using an enhanced chemiluminescence detection system (Tanon). Adobe Photoshop software was used to analyze protein expression levels after measuring the protein band intensity.

### 4.8 Immunofluorescence staining and immunohistochemistry staining

For immunofluorescence staining, the sections were treated using heat underwent heat mediated antigen retrieval with sodium citrate buffer (pH of 6.0) for 16 min, then blocked in 10% normal donkey serum (YEASON) for 1 h. Primary antibodies were diluted in PBS and incubated with sections in a humidified chamber for 16 h at 4°C. Then the sections were incubated with the corresponding secondary antibody conjugated to FITC or TRITC (YEASON) for 1 h at 37°C, and the sections were stained with hoechst for the detection of nuclei as well. Representative microphotographs were acquired using a Nikon laser scanning confocal microscope (Nikon) and analyzed by NIS‐Elements Viewer imaging software. The sections after the acquisition of images can be re‐incubated with new primary antibodies after antigen repair to achieve simultaneous labeling of different molecules. For immunohistochemistry staining, the sections after immunofluorescence staining were underwent hydrogen peroxide blocking to inhibit endogenous peroxidase activity. After incubation with HRP‐conjugated secondary antibodies (ZSGB‐BIO), the sections were visualized with DAB and restaining with hematoxylin to label nuclei. Images were obtained through VENTANA DP200 (Roche).

### 4.9 RNA‐seq data processing

Ovarian samples were collected and performed RNA‐seq analysis by Personal Biotechnology (Shanghai, China). Genes with |log2FC| > 1 and *p* value < 0.05 were considered differential expression genes (DEGs). Heat maps generated by the R package pheatmap were used to visualize the expression patterns of DEGs in different groups. Gene ontology enrichment and Kyoto Encyclopedia of Genes and Genomes (KEGG) analyses were performed by the R package cluster Profiler. Gene ontology enrichment terms and KEGG pathways with a *p* value < 0.05 were considered statistically significant and are shown by dot plots. To compare the differences in biological process signaling pathways between different groups, Gene Set Enrichment Analysis (GSEA) was performed by the R package cluster Profiler. Only gene sets with *p* value < 0.05 were shown by using the R package enrich plot.

### 4.10 Electron microscopy

For mitochondrial ultrastructure analysis, ovaries were fixed for 12 h at 4°C in 2.5% glutaraldehyde. After being rinsed in PBS, ovaries were postfixed in 2% ferrocyanide‐reduced osmium tetroxide, stained with 1% uranyl acetate aqueous solution, dehydrated in ascending concentrations of ethanol, passed through acetone and finally embedded in Epon resin. Ultrathin sections were then counterstained with 2% uranyl acetate and lead citrate. Samples were analyzed using a transmission electron microscope (HITACHI).

### 4.11 Protein mass spectrometry

Total protein was extracted with cell lysis buffer (WIP, Beyotime) containing PMSF (1:100, Beyotime) and protein levels were determined by the Mass Spectrometry Lab of China Agricultural University (Beijing, China). Proteins with |log2FC| > 1 and adjusted *p* value (by the Benjamini‐Hochberg method) < 0.05 were considered differential proteins. Gene ontology enrichment analyses were performed by the R package cluster Profiler. Heat maps generated by the R package pheatmap were used to visualize the expression patterns differentially proteins in different groups.

### 4.12 Cleavage under targets and Tagmentation (CUT & tag)

The CUT & Tag assays were performed using a Hieff NGS® G‐Type In‐Situ DNA Binding Profiling Library Prep Kit (12598ES; YEASEN, China) according to the manufacturer's protocol. Briefly, NGOs were isolated and meticulously prepared according to the aforementioned protocols, ensuring a minimum cell count exceeding 200 units, and were then subjected to a series of optimized premixed buffer solutions for cell capture using Con A magnetic beads. Specific primary and secondary antibodies targeting FOXO3 were applied to promote protein binding, followed by transposase treatment to effectuate DNA‐protein crosslinking. After Proteinase K digestion, DNA fragments were carefully recovered and purified using DNA Selection Beads. The purified DNA was subsequently amplified via PCR with specific primers and indexs, with a set cycle number of 16, yielding a final product concentration greater than 18 ng/μL. The amplified library was further refined through purification prior to sequencing by Personal Biotechnology (Shanghai, China). Postsequencing, the data were aligned to the mouse genome reference (GRCm39), and a KEGG enrichment analysis was performed on nearest genes to the identified peaks, thereby elucidating the biological processes and gene types regulated by FOXO3 within NGOs.

### 4.13 Statistics and reproducibility

All statistical data from at least three independent experiments are presented as mean ± s.e.m. or s.d. unless otherwise stated. And the number of samples used in each group is labeled in parentheses as (*n*). Data were analyzed by a two‐tailed unpaired student *t* test which is provided by GraphPad Prism 8 statistical software. All graphs of the immunoblotting were obtained from three independent replicate experiments.

## AUTHOR CONTRIBUTIONS

C.W. conceived and supervised the study. S.Q., X.C., and C.W. designed the experiments. C.C., T.Z., M.H., M.G., T.Z., J.Z., L.Z., W.Z., Z.C., W.W., B.Z., and G.X., performed and analyzed the experiments. S.Q., X.C., Z.Z., C.C., and C.W. wrote the manuscript. All the authors read and approved the manuscript.

## FUNDING INFORMATION

This work was supported by the National Key Research & Developmental Program of China (2023YFD1300501, 2022YFC2703803, 2018YFC1003700, 2018YFC1003801), the National Natural Science Foundation of China (32,371,167, 32,071,132, 31,872,792, 32,270,904, 32,070,839), and the Innovative Project of State Key Laboratory of Animal Biotech Breeding (2024SKLAB 1‐1).

## CONFLICT OF INTEREST STATEMENT

The authors declare that there are no conflicts of interest regarding the publication of this paper. This includes, but is not limited to, financial, personal, or professional affiliations that could be perceived to influence the content or interpretation of the findings within this manuscript.

## Supporting information


Figures S1–S3.

## Data Availability

The data that support the findings of this study are available from the corresponding author upon reasonable request.
