# Supplementary material for: Oocytes maintain low ROS levels to support the dormancy of primordial follicles
Source: Aging Cell. 2024 Sep 19;24(1):e14338. doi: 10.1111/acel.14338 (PMC11709087; doi:10.1111/acel.14338)
Supplement: Supplementary file 1 — Figures S1–S3. [file ACEL-24-e14338-s001.doc]

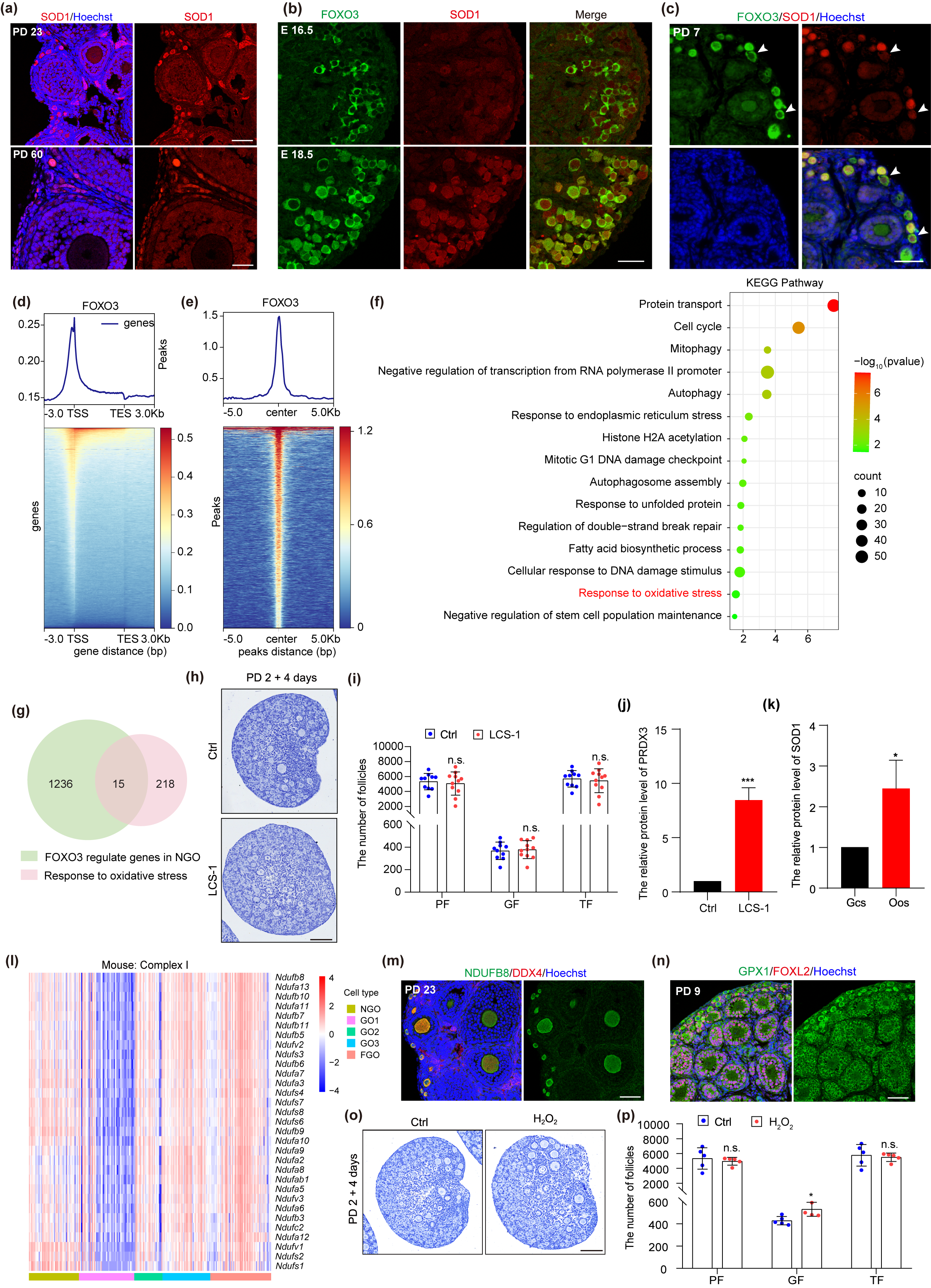


**FIGURE S1 NGO maintains dormancy due to higher level of SOD1** (a) Representative images of immunofluorescence staining for SOD1 (red) in ovary tissues of puberty mice (PD 23) and adult mice (PD 60). The nuclei were stained with hoechst (blue). Scale bar, 100 μm. (b) Immunofluorescence staining for SOD1 (red) and FOXO3 (green) in embryonic (E) 16.5 and E 18.5 mouse ovaries (PFs are forming). Scale bar, 50 μm. (c) Immunofluorescence staining for SOD1 (red) and FOXO3 (green) in PD 7 mouse ovaries (PFs are activating). The nuclei were stained with hoechst. Scale bar, 50 μm. (d) The number of FOXO3 combination genes in a ± 3 kb window of TSSs. (e) The number of FOXO3 combination peaks in a ± 5 kb window of TSSs. (f) Pathways enrichment using KEGG analysis of FOXO3-enriched genes in NGOs. *P* value < 0.05, FDR < 0.25. (g) A Venn diagram illustrates the overlap between genes enriched on the FOXO3 promoter and those involved in the oxidative stress response pathway, including *Jun*, *Cpeb2*, *Etv5*, *Sesn2*, *Pnpla8*, *Vkorc1l1*, *Sod2*, *Prdx1*, *Prdx3*, *Mapk9*, *Atg7*, *Htra2*, *Fer*, *Nfe2l1* and *Hdac2*. (h) Representative images of sections from PD 2 ovaries cultured for 4 days with treatment of 5 μM LCS-1 or not. Scale bar, 100 μm. (i) Numbers of follicles at different developmental stages were counted in each ovarian after treatment with LCS-1 or not (n = 10). (j) The relative expression level of non-reducing PRDX3 to β-Tubulin in ovaries treated with LCS-1 or not. (k) The relative expression level of SOD1 in oocytes and granulosa cells to β-Actin of PD 5 mouse ovaries. (l) Heatmap illustration displays differential genes in complex I from oocytes at different developmental stages. Original analysis data came from GSE107746. (m) Representative images of immunofluorescence staining for NDUFB8 (green) in ovary tissues of PD 23 mice. Oocytes were stained with DDX4 (red) and the nuclei were stained with hoechst (blue). Scale bar, 100 μm. (n) Representative images of immunofluorescence staining for GPX1 (green) in ovary tissues of PD 9 mice. GC were stained with FOXL2 (red) and the nuclei were stained with hoechst (blue). Scale bar, 100 μm. (o) Representative images of sections from PD 2 ovaries cultured for 4 days with treatment of 1 mM H_2_O_2_ or not. Scale bar, 100 μm. (p) Numbers of follicles at different developmental stages were counted in each ovarian after treatment with H_2_O_2_ or not (n = 5). ****p* < 0.001, **p* < 0.05, n.s., not significant.


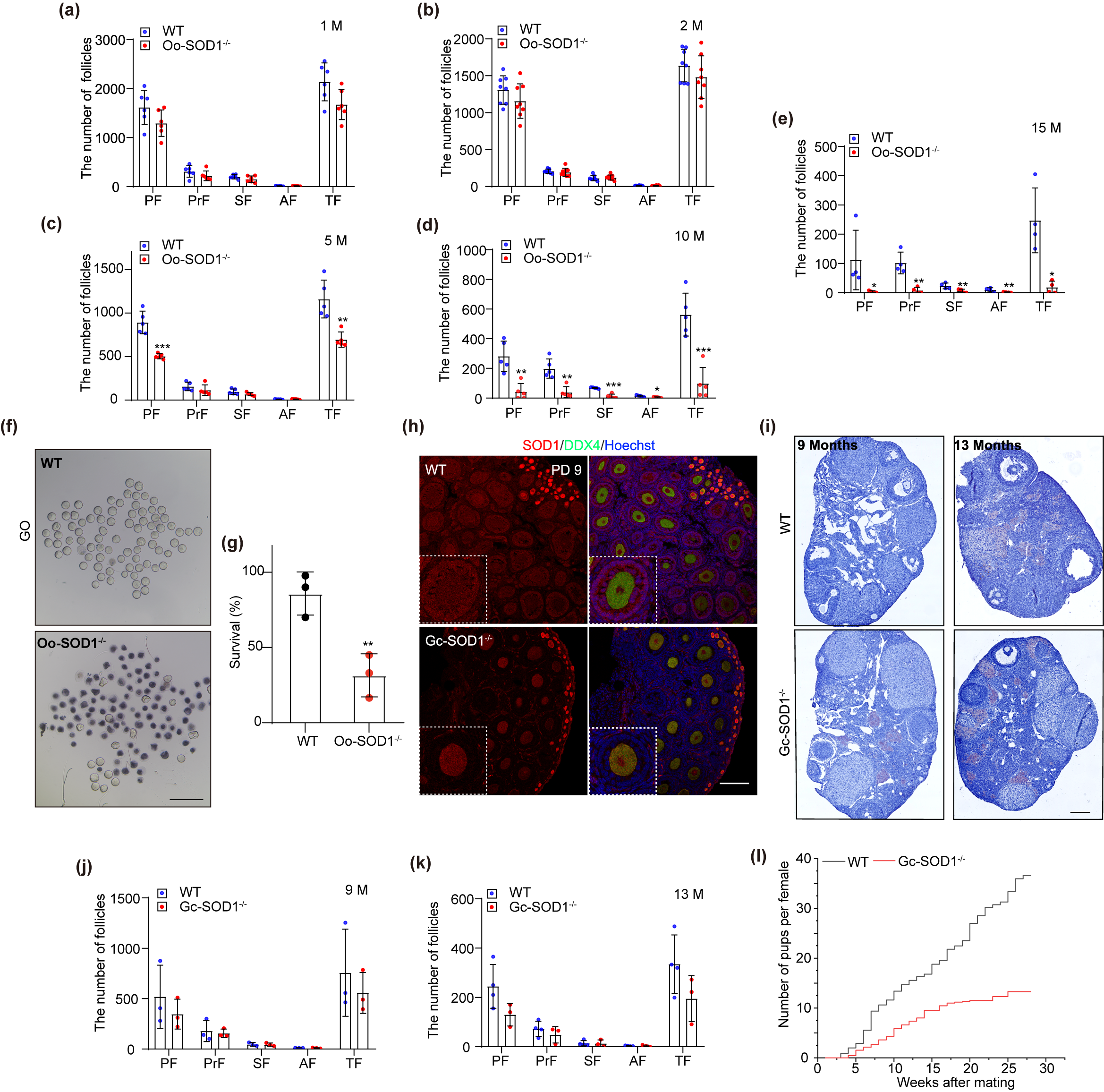


**FIGURE S2 The role of SOD1 in maintaining PFs shows age dependency.** (a, b, c, d, e) Numbers of follicles at different developmental stages were counted in each ovary from WT and Oo-SOD1^-/-^ mice at the age of 1 month (a, n = 6), 2 months (b, n = 8), 5 months (c, n = 5), 10 months (d, n = 5) and 15 months (e, n = 4), respectively. (f, g) The survival rate of GO from WT and Oo-SOD1^-/-^ mice measured by Trypan blue staining. Scale bar, 100 μm (n = 3). (h) Immunofluorescence staining for SOD1 (red) was used to detect knockout efficiency in PD 9 granulosa cells. Oocytes were stained with DDX4 (green) and the nuclei were stained with hoechst (blue). Scale bar, 100 μm. (i) Representative images of ovarian sections from WT and Gc-SOD1^-/-^ mice at the age of 9 months and 13 months. Scale bars, 200 μm. (j, k) Numbers of follicles at different developmental stages were counted in each ovary from 9-month-old mice (j, n = 4) and 13-month-old mice (k, n = 3). There was no significant difference between the groups. (l) Fertility testing of WT and Gc-SOD1^-/-^ female mice (n = 16). ****p* < 0.001, ***p* < 0.01, **p* < 0.05.


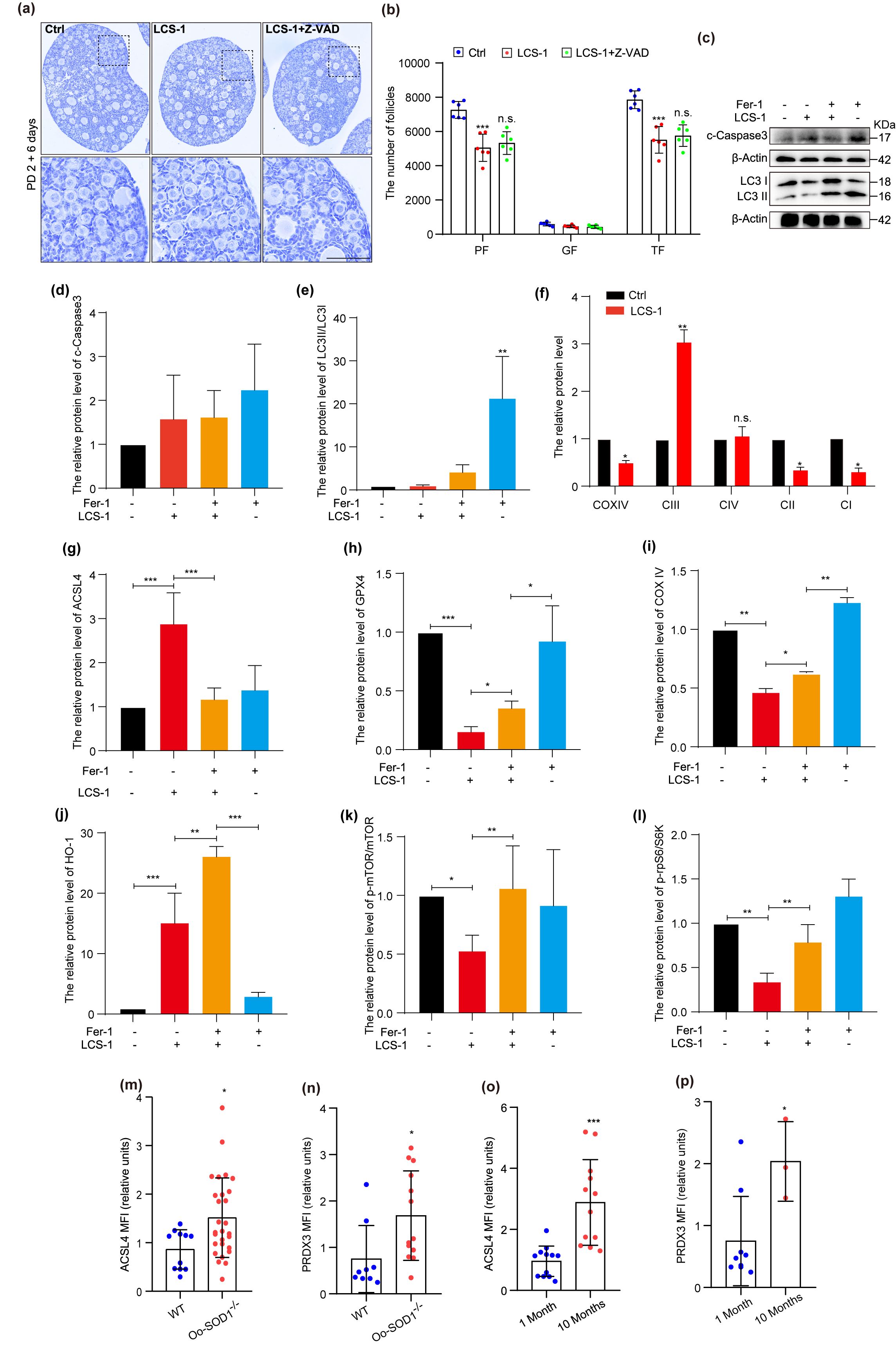


**FIGURE S3 Ferroptosis may be the main mechanism leading to the loss of PFs in mice.** (a) Representative images of sections from PD 2 ovaries cultured for 6 days with treatment of LCS-1 and 10 μM Z-VAD (Caspase inhibitors) or not respectively. Scale bar, 100 μm. (b) Numbers of follicles at different developmental stages were counted in each ovary (n = 6). (c) Immunoblotting of proteins associated with apoptosis and autophagy pathway in ovaries treated with LCS-1 and Fer-1 or not. (d, e) The relative expression level of c-caspase3 (d) and LC3Ⅱ/LC3Ⅰ (e) in ovaries treated with LCS-1 and Fer-1 or not. (f) The relative expression level of COXⅣ, CⅢ, CⅣ, CⅡ, CⅠ to β-Actin in ovaries treated with LCS-1 or not. (g, h, i, j, k, l) The relative expression level of ACSL4 (g), GPX4 (h), COXⅣ (i), HO-1 (l), p-mTOR/mTOR (m) and p-rpS6/S6K (n) in ovaries treated with LCS-1 and Fer-1 or not. (m, n) The mean fluorescence intensity of ACSL4 (m) and PRDX3 (n) in PFs from WT and Oo-SOD1^-/-^ mice (n = 3). (o, p) The mean fluorescence intensity of ACSL4 (o) and PRDX3 (p) in PFs from 1-month-old and 10-month-old mice (n = 3). ****p* < 0.001, ***p* < 0.01, **p* < 0.05, n.s., not significant.
